# Identification of QTNs Controlling 100-Seed Weight in Soybean Using Multilocus Genome-Wide Association Studies

**DOI:** 10.3389/fgene.2020.00689

**Published:** 2020-07-16

**Authors:** Zhongying Qi, Jie Song, Kaixin Zhang, Shulin Liu, Xiaocui Tian, Yue Wang, Yanlong Fang, Xiyu Li, Jiajing Wang, Chang Yang, Sitong Jiang, Xu Sun, Zhixi Tian, Wenxia Li, Hailong Ning

**Affiliations:** ^1^Key Laboratory of Soybean Biology, Ministry of Education, Key Laboratory of Soybean Biology and Breeding/Genetics, Ministry of Agriculture, Northeast Agricultural University, Harbin, China; ^2^State Key Laboratory of Plant Cell and Chromosome Engineering, Institute of Genetics and Developmental Biology, Chinese Academy of Sciences, Beijing, China

**Keywords:** soybean, hundred-seed weight, multilocus GWAS, QTNs, four-way RILs

## Abstract

Hundred-seed weight (HSW) is an important measure of yield and a useful indicator to monitor the inheritance of quantitative traits affected by genotype and environmental conditions. To identify quantitative trait nucleotides (QTNs) and mine genes useful for breeding high-yielding and high-quality soybean (*Glycine max*) cultivars, we conducted a multilocus genome-wide association study (GWAS) on HSW of soybean based on phenotypic data from 20 different environments and genotypic data for 109,676 single-nucleotide polymorphisms (SNPs) in 144 four-way recombinant inbred lines. Using five multilocus GWAS methods, we identified 118 QTNs controlling HSW. Among these, 31 common QTNs were detected by various methods or across multiple environments. Pathway analysis identified three potential candidate genes associated with HSW in soybean. We used allele information to study the common QTNs in 20 large-seed and 20 small-seed lines and identified a higher percentage of superior alleles in the large-seed lines than in small-seed lines. These observations will contribute to construct the gene networks controlling HSW in soybean, which can improve the genetic understanding of HSW, and provide assistance for molecular breeding of soybean large-seed varieties.

## Introduction

Soybean [*Glycine max* (L.) Merr.] is an important source of edible oil and plant protein for humans. Soy-based foods are popular in the international market, and the demand for soybeans is increasing. Hundred-seed weight (HSW) is an important agronomic trait related to soybean yield and food quality. Therefore, locating the quantitative trait locus (QTLs) related to HSW and exploring valuable alleles associated with the QTLs have important theoretical and application value for improving soybean yield and quality.

Linkage analysis based on framework linkage maps of low polymorphism molecular markers, such as restriction fragment length polymorphisms (RFLPs) and simple sequence repeats (SSRs), is the main method for detecting QTLs controlling HSW in soybean. Quantitative trait loci associated with HSW have been identified in F_2_ (Wang et al., 2010), recombinant inbred line (RIL; Reinprecht et al., 2006), and backcross (BC; Li W.X. et al., 2008) populations using QTL mapping methods such as interval mapping (IM; Lander and Botstein, 1989), composite interval mapping (CIM; Zeng, 1994), and inclusive complete interval mapping (Wang, 2009). Genome-wide CIM (Wang et al., 2016b; Wen et al., 2017) was recently proposed for detecting small-effect and linked QTLs. However, QTLs detected by these methods have rarely been applied successfully to marker-assisted breeding research because large linkage between markers and QTL is feasible to be interrupted. Thus, genome-wide association studies (GWASs) have been conducted gradually for QTL mapping of HSW in soybean.

Drifting and selection of genes during crop breeding and domestication produce complex population structures, which can cause false positives. Statistical methods to circumvent this issue include case–control studies, transmission imbalance tests, structured associations, principal component analysis (PCA), and hybrid models. A newly developed GWAS statistical method based on a mixed linear model (MLM) can overcome the above difficulties and address fixed and random effects flexibly. Common variables from STRUCTURE (Pritchard et al., 2000) and PCA can serve as fixed effects to account for false associations due to group structure. The complex relationship between individuals constitutes the affinity matrix in the MLM (Yu et al., 2006). The kinship matrix derived from a set of aggregated individuals is used in the computationally efficient compressed MLM (CMLM; Zhang et al., 2010). In some cases, the traditional maximum-likelihood method cannot be used to solve an MLM with a large number of genotypes because the calculation intensity is too large. Therefore, the efficient mixed model association (EMMA; Kang et al., 2008) algorithm was developed to reset the parameter of MLM likelihood function. Among the many software packages available, mrMLM. GUI integrates the most accurate and computationally efficient methods for GWAS and genome prediction selection, and these methods are integrated into the R package, which can analyze large amounts of data in the shortest time.

Genome-wide association study has been used to detect QTLs related to HSW in soybean. Zhou et al. (2015) used a total of 302 germplasm accessions including wild soybean varieties, local varieties, and bred cultivars to detect HSW loci on chromosome 17, while Zhang et al. (2016) used 309 soybean germplasm accessions to detect 22 HSW QTLs by GWAS analysis. These studies used single-gene locus methods such as MLM (Yu et al., 2006; Zhang et al., 2010). The mrMLM method used in our study is a multilocus method that detects more small-effect QTLs than the single-locus method (He et al., 2019), reducing the probability of false positives (Fang et al., 2017).

We used 144 RILs with four-way hybridization (FW-RILs) to obtain phenotypic data for the HSW and SNP genotype data across multiple environments. The multilocus GWAS method was used to detect quantitative trait nucleotides (QTNs) associated with HSW. We identified potential candidate genes and common QTNs associated with large-seed lines across multiple methods or in multiple environments, laying the foundation for high-yield soybean breeding.

## Materials and Methods

### Plant Materials

In 2008, the four soybean varieties Kenfeng 14 (HSW 21 g), Kenfeng 15 (HSW 18 g), Kenfeng 19 (HSW 19 g), and Heinong 48 (HSW 25 g) were used to prepare double hybrid combinations. Kenfeng 14, Kenfeng 15, Kenfeng 19, and Heinong 48 were derived from the crosses Suinong 10 × Changnong 5, Suinong 14 × Kenjiao 9307, Hefeng 25 × (Kenfeng 4 × Gong 8861-0), and Ha 90-6719 × Sui 90-5888, respectively. In 2009, F_1_ (Kenfeng 14 × Kenfeng 15) was used as the female parent, and F_1_ (Kenfeng 19 × Heinong 48) was used as the male parent to produce four-way hybrid F_1_ seeds. From 2010 to 2012, the seeds were planted in Harbin in the summer and in Hainan in the winter. Four-way RILs (144 lines) were obtained by continuous self-crossing for six generations using the single-seed descent method.

### Field Experiments and Phenotypic Data Collection

The 144 FW-RILs were planted in 20 environments; details of the planting environments are summarized in Supplementary Table S1. The field experiment in each environment followed a randomized block design. Plot length was 5 m, ridge distance was 65 cm, plant spacing was 6 cm, three rows were repeated three times, and field management was in accordance with general field cultivation. After maturity, 10 plants showing uniform growth were selected from each row, and HSW was measured indoors. Phenotypic values are all averages of three replicates.

### Phenotypic Variation Analysis

Mean, variance, standard deviation, minimum, maximum, skewness, and kurtosis of FW-RILs were calculated in each environment. Analysis of variance was performed on phenotypic data from the 20 environments using the following model:

$$x_{i\,j}=\mu +G_{i}+E_{j}+G\,E_{i\,j}+\varepsilon_{i\,j}$$

where μ is the grand average, *G*_*i*_ is the *i*th genotype effect, *E*_*j*_ is the *j*th environment effect, *GE*_*ij*_ is the genotype × environmental effect, and ε*_*ij*_* is the error effect following N(0, σ^2^).

The mean squared difference was estimated by applying the mean square of each variation source, and the estimated generalized heritability was calculated using the following formula.

$$h^{2}=\frac{\sigma_{G}^{2}}{\sigma_{G}^{2}+\frac{\sigma_{G\,E}^{2}}{e}+\frac{\sigma^{2}}{e\,r}}$$

where *h*^2^ is the generalized heritability of gene × environment interaction,$\sigma_{G}^{2}$ is the genotype variance, $\sigma_{G\,E}^{2}$ is the genotype × environmental interaction variance, σ^2^ is the error variance, *e* is the number of environments, and *r* is the number of repetitions in each environment. Data were analyzed using the general linear model method in Statistical Analysis System (SAS) 9.2, North Carolina.

### Genotyping

Leaves of each accession were collected in the field at the three-leaf stage and placed in 2-mL centrifuge tubes. Liquid nitrogen was added to the tubes, and leaves were rapidly ground to a white powder. Soybean genomic DNA was extracted using the CTAB method (Doyle et al., 1990). DNA was dissolved in 50 μL ddH_2_O, 1 μL RNAase (10 mg/mL) was added, and samples were stored at −20°C. Single-nucleotide polymorphism genotyping was performed by Beijing Boao Biotechnology Co., Ltd. (Beijing, China) using the SoySNP660K BeadChip. A total of 109,676 SNPs were obtained on 20 chromosomes after mass filtration based on the following criteria: minor allele frequency (MAF) of the SNP was identified (MAF > 0.05), maximum missing data rate <10% (Belamkar et al., 2016), and heterozygous loci deleted.

### Analysis of Linkage Disequilibrium and Population Structure

Linkage disequilibrium (LD) analysis was performed using TASSEL 5.0 (Bradbury et al., 2007) to calculate the square of the allelic frequency dependence (*r*^2^) of all SNPs located within 10-Mb physical distance, and the physical distance of LD decay was estimated as the position where *r*^2^ dropped to half of its maximum value.

Population structure was analyzed after filtration on 5,000 SNPs evenly distributed on 20 chromosomes using STRUCTURE 2.3.4 (Pritchard et al., 2000). The number of burn-in iterations per run was 100,000, followed by 100,000 Markov Chain Monte Carlo replications after burn-in. Mixed and allele frequency correlation models were considered in the analysis. Five implicit iterations were used in the STRUCTURE analysis. The best subgroups (K) were identified according to the method of Evanno et al. (2005) using STRUCTURE HARVESTER (Earl and Vonholdt, 2012). The assumed number of *K* ranged from 1 to 10.

### Genome-Wide Association Studies

Genome-wide association studies were performed using the software mrMLM. GUI (version 3.0). There are five multilocus GWAS methods in the mrMLM package that can be used to identify significant QTNs, namely, mrMLM (Wang et al., 2016a), FASTmrMLM (Tamba and Zhang, 2018), FASTmrEMMA (Wen et al., 2018), pLARmEB (Zhang J. et al., 2017; Zhang Y. et al., 2017), and ISIS EM-BLASSO (Tamba et al., 2017). In the first stage, the critical *P* value parameter was set to 0.01, except that of FASTmrEMMA was set to 0.005. In the final stage, the critical LOD value of significant QTNs was set to 3. All matrices involved in these five methods were calculated using mrMLM.GUI3.0.

### Superior Allele Analysis

Among the significant QTNs identified, some were detected in a variety of environments or methods. These significant QTNs are referred to as common QTNs. The positive and negative of the QTN effect values were used as the criterion for selecting superior alleles. If the QTN effect value is positive, the genotype of code 1, which was obtained by GWAS, is the superior allele; if the QTN effect value is negative, the other genotype is the superior allele. The percentage of excellent alleles is calculated as follows: for each line, the percentage of excellent alleles is the number of common QTNs carrying excellent alleles divided by the total number of common QTNs; for each common QTN, the percentage of alleles among the 144 FW-RILs is equal to the number of rows containing the alleles divided by the total number of rows. Heat maps were generated using the R package Complexheatmap (Mellbye and Schuster, 2014).

### Identification of Potential Candidate Genes

The physical locations of generic QTNs identified by the above methods were marked. Intervals containing each of the common QTNs were then selected on the Phytozome website. These intervals are determined by the LD decay rate. Highly expressed genes at certain locations based on relevant traits were selected. This step was done on the BAR website. Finally, the genes were matched to those in the Kyoto Gene and Genomics Encyclopedia (KEGG) to analyze gene expression pathways and identify potential candidate genes.

## Results

### Phenotypic Variation

We measured HSW phenotypic data for 144 FW-RILs and the four parent lines in 20 environments. The phenotypic values for the four parents are given in Supplementary Table S2. In 19 environments, the kurtosis and skewness (absolute value) were less than 1, indicating a continuous normal distribution of HSW (Supplementary Table S3 and Figure S1). We used SAS software to perform a descriptive statistical analysis, conduct an analysis of variance, and estimate the generalized heritability of phenotypic values (Table 1). The results showed that significant phenotypic variation was detected in the HSW of 144 FW-RILs across 20 environments. An analysis of variance revealed significant differences in genotype effects, environmental effects, and genotype × environment interactions (Table 1). Therefore, HSW is not only affected by genotype and environmental factors, but also by genotype-by-environmental interaction effects. The heritability of HSW in multiple environments was 79%. This indicates that although HSW is affected by the environment, most of the variation is due to genetic effects.

**TABLE 1:** Joint ANOVA of HSW of FW-RILs in multiple environments and heritability.

**Source**	**DF**	**SS**	**MS**	***F***	**Pr > *F***	**Variance component**
Replication	2	0.0060	0.0030	1.43	0.2390	–
Environment	19	12,262.04	645.37	308,403	<0.0001	1.72
Genotype	143	10,032.07	70.15	33,524.6	<0.0001	1.02
Genotype × environment	2,385	38,569.94	16.17	7,728.05	<0.0001	5.39
Error	5,094	10.66	0.0021	–	–	0.0021
*h*^2^	–	–	–	–	–	0.79

### Population Structure

**FIGURE 1:**
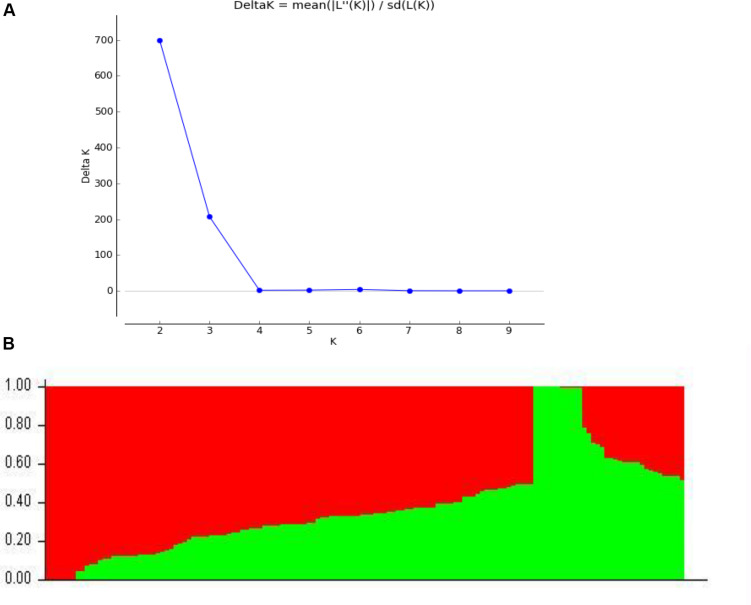
Population structure based on 5,000 SNPs distributed across 20 chromosomes. **(A)** Plot of 1K calculated for *K* = 1−10. **(B)** Population structure (*K* = 2); the areas of the two colors (green and red) illustrate the proportion of each subgroup.

Among 109,676 SNPs, we selected 5,000 SNPs with superior polymorphism evenly distributed on the 20 soybean chromosomes. We used STRUCTURE 2.3.4 to calculate Δ*K* (Figure 1A; *K* = 1-10) and identified two subgroups (selected *K* = 2) based on the Δ*K* value (Figure 1B). We analyzed the *r*^2^ values of all pairs of SNPs located within 10 Mb of each other and determined the LD decay trend based on regression to the negative natural logarithm.

### QTNs Detected by Multilocus GWAS Methods

**FIGURE 2:**
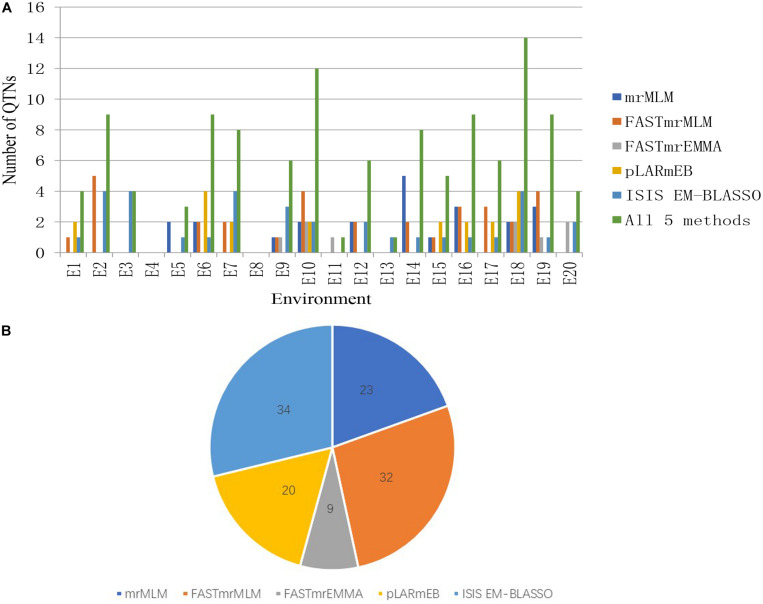
**(A)** The total numbers of significant QTNs detected in 20 environments across five methods. **(B)** The total numbers of significant QTNs detected using each of five multilocus GWAS methods in 20 environments.

We detected 23, 32, 9, 20, and 34 significant QTNs using the mrMLM, FASTmrMLM, FASTmrEMMA, pLARmEB, and ISIS EM-BLASSO methods, respectively, with 4, 9, 4, 0, 3, 9, 9, 0, 6, 12, 1, 6, 1, 8, 5, 9, 6, 14, 9, and 4 significant QTNs detected in each of 20 environments, respectively. No significant QTNs were detected in E4 or E8 (Figure 2 and Supplementary Table S4).

We further examined significant QTNs occurring in multiple environments. Three common QTNs were identified in E2 and E3 (Table 2). AX-157525995 was located on chromosome 6, with LOD values ranging from 3.08 to 4.48 (Table 2). This QTN explained a proportion of phenotypic variance (PVE) of between 3.28 and 6.43%. AX-157369770 was located on chromosome 11, with LOD values ranging from 3.07 to 3.80 (Table 2). This QTN explained a proportion of phenotypic variance (PVE) of between 3.78 and 5.02% (Table 2). AX-157588392 was located on chromosome 12, with LOD values ranging from 4.20 to 4.26 (Table 2). This QTN explained a proportion of phenotypic variance (PVE) of between 6.36 and 6.64% (Table 2). The QTN effect direction (positive or negative) was consistent across environments and methods (Table 2).

**TABLE 2:** Stable expressed QTNs identified in multiple environments and by multiple methods.

**Method^a^**	**Env**	**Marker**	**Chr**	**Marker position**	**QTN effect**	**LOD score**	***r*^2^ (%)^b^**
5, 5	E2, E3	*AX-157525995*	6	51088854	0.66, 0.74	3.08, 3.90	3.28, 4.24
2, 5	E2, E2	–	–	–	0.66, 0.92	3.08, 4.48	3.28, 6.43
2, 5	E2, E3	–	–	–	0.74, 0.92	3.90, 4.48	4.24, 6.43
2, 5	E2, E3	*AX-157369770*	11	32617288	−0.87, −0.75	3.80, 3.07	5.02, 3.78
2, 5	E2, E3	*AX-157588392*	12	17219655	−1.01, −0.94	4.20, 4.26	6.64, 6.36

*^a^mrMLM, FASTmrMLM, FASTmrEMMA, pLARmEB, and ISIS EM-BLASSO were indicated by 1–5, respectively. ^b^*r*^2^ (%), proportion of total phenotypic variation explained by each QTN.*

By comparing the results of different methods, 31 common QTNs were detected by two or more methods (Table 3); these were located on chromosomes 2, 3, 4, 6, 7, 8, 9, 10, 11, 12, 14, 15, 16, 18, and 20. Their LOD values ranged from 3.00 to 6.68, and the PVE ranged from 3.53E-09 to 15.07. The direction of action (positive or negative) for each universal QTN was also consistent across the different methods (Table 3).

**TABLE 3:** Common QTNs for hundred-seed weight in soybean across different multilocus method.

**Method^a^**	**Marker**	**Chromosome**	**Position (bp)**	**QTN effect**	**LOD score**	***r*^2^ (%)^b^**
1, 2, 3, 4, 5	*AX-116905453*	2	44205396	1.43, 1.23, 2.23, 1.13, 1.15	5.43, 5.43, 4.64, 5.26, 5.48	15.07, 12.14, 8.42, 10.12, 10.74
3, 5	***AX-157257435***	**2**	**43283682**	**2.05**, **0.70**	**3.32**, **3.22**	**12.83**, **5.91**
1, 2, 5	*AX-157170687*	3	1393672	0.72, 0.56, 0.54	3.08, 3.98, 3.30	12.40, 7.52, 6.94
1, 4, 5	*AX-157052803*	3	1722938	0.99, 0.90, 0.86	4.38, 4.95, 3.86	12.96, 10.80, 10.08
1, 2	***AX-157407598***	**3**	**39988963**	**0.90**, **0.61**	**3.52**, **3.52**	**9.03**, **4.59**
1, 2, 4, 5	*AX-116924879*	3	5093950	−0.82, −0.60, −0.60, −0.73	3.16, 3.70, 4.13, 4.33	11.33, 6.23, 6.23, 8.96
2, 5	***AX-157528951***	**3**	**6197954**	−**0.66**, −**0.94**	**3.51**, **6.68**	**5.84**, **11.04**
1, 2, 3, 4, 5	***AX-157442983***	**4**	**6484678**	**1.15**, **0.88**, **1.98**, **0.95**, **0.92**	**4.00**, **3.02**, **3.38**, **3.56**, **3.77**	**12.52**, **7.33**, **10.44**, **8.03**, **7.98**
1, 2, 4, 5	*AX-157126994*	6	49959758	−0.99, −0.83, −0.78, −0.64	5.67, 5.67, 5.31, 3.87	14.03, 10.74, 9.52, 6.35
2, 5	*AX-157525995*	6	51088854	0.92, 0.66 (0.74)	4.48, 3.08 (3.90)	6.43, 3.28 (4.24)
1, 2, 5	*AX-157457247*	7	3254514	−0.71, −0.49, −0.52	4.81, 3.23, 3.66	10.00, 5.06, 5.72
2, 5	*AX-157395672*	7	37249194	−0.77, −0.82	3.67, 4.26	4.49, 5.13
1, 2	***AX-157388275***	**8**	**46069989**	−**0.88**, −**0.70**	**4.08**, **4.08**	**11.96**, **7.74**
1, 2	*AX-157555217*	8	47740465	0.95, 0.87	3.85, 4.22	13.46, 11.42
4, 5	***AX-157129457***	**9**	**40507825**	**0.59**, **0.59**	**3.71**, **3.13**	**3.80**, **3.82**
1, 2, 3, 4, 5	*AX-157355369*	10	49273669	0.90, 0.63, 1.25, 0.70, 0.56	3.52, 3.52, 3.70, 5.02, 3.00	12.43, 6.50, 6.00, 8.03, 5.19
1, 2	*AX-157480162*	10	50172467	0.85, 0.60	3.14, 3.12	8.82, 4.66
2, 5	***AX-157369770***	**11**	**32617288**	−**0.87**, −**0.75**	**3.80**, **3.07**	**5.02**, **3.78**
2, 5	*AX-157588392*	12	17219655	−1.01, −0.94	4.20, 4.26	6.64, 6.36
3, 5	***AX-157090659***	**14**	**881999**	**1.57**, **0.80**	**3.28**, **3.01**	**4.80**, **5.21**
3, 5	*AX-157446872*	14	37252443	−1.75, −0.88	3.86, 4.15	6.61, 7.80
2, 3, 4	***AX-157439837***	**15**	**12672673**	**1.16E-05**, **1.01**, **0.46**	**3.60**, **3.45**, **3.22**	**3.53E-09**, **6.34**, **5.15**
1, 2	*AX-157069540*	16	37223445	−0.67, −0.55	4.17, 4.17	10.06, 6.91
2, 5	*AX-157575017*	16	32946305	−0.43, −0.45	3.06, 3.24	4.35, 4.78
1, 2, 4	*AX-157516116*	16	33525379	−0.72, −0.55, −0.55	3.35, 4.44, 5.65	10.30, 6.08, 6.09
2, 4	*AX-157553398*	18	56507541	0.48, 0.49	3.52, 3.05	4.43, 4.63
1, 2, 4	*AX-157268182*	18	56589017	−0.81, −0.60, −0.54	3.89, 3.89, 3.48	9.95, 6.05, 4.92
1, 2	*AX-157352830*	18	56836743	0.89, 0.71	3.75, 4.55	11.44, 7.76
1, 2	***AX-157467282***	**20**	**43174088**	−**0.77**, −**0.43**	**4.71**, **3.67**	**11.61**, **3.93**
1, 2	*AX-116884145*	20	47162277	−0.75, −0.50	3.19, 3.01	8.45, 3.94
2, 5	***AX-157324617***	**20**	**42347884**	**0.54**, **0.66**	**3.31**, **3.73**	**5.35**, **7.38**

*^a^mrMLM, FASTmrMLM, FASTmrEMMA, pLARmEB, and ISIS EM-BLASSO were indicated by 1–5, respectively. ^b^*r*^2^ (%), proportion of total phenotypic variation explained by each QTN. Bold text indicates the QTNs appeared to be near QTLs associated with hundred-seed weight in soybean that had been mapped in earlier studies.*

**FIGURE 3:**
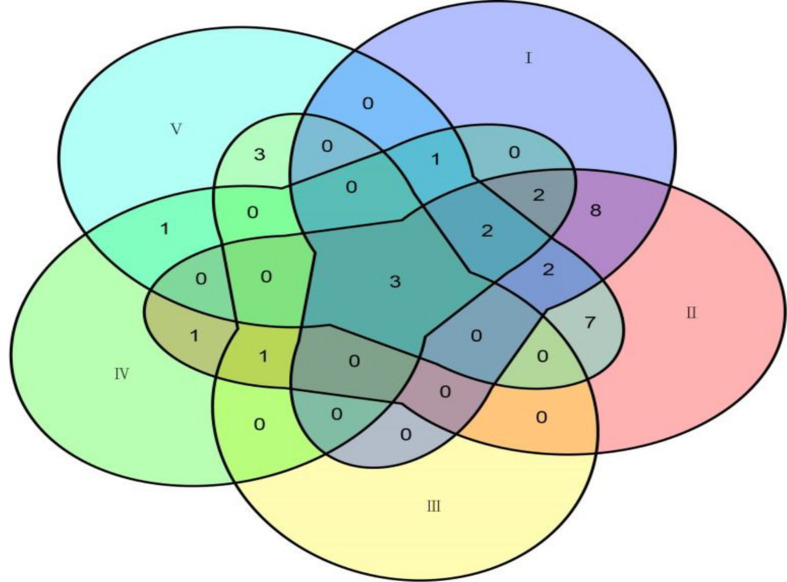
The connection of common QTNs detected by five methods. Method numbers correspond to (I) mrMLM, (II) FASTmrMLM, (III) FASTmrEMMA, (IV) pLARmEB, and (V) ISIS EM-BLASSO.

**FIGURE 4:**
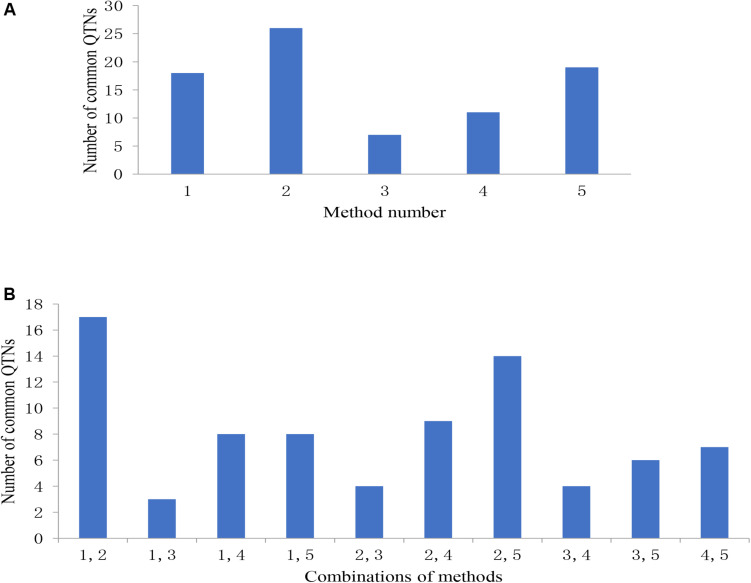
**(A)** The number of common QTNs detected by different methods and **(B)** different combinations of methods. Method numbers correspond to (1) mrMLM, (2) FASTmrMLM, (3) FASTmrEMMA, (4) pLARmEB, and (5) ISIS EM-BLASSO.

Among the 31 common QTNs, 20, 6, 2, and 3 QTNs were detected commonly by two, three, four, and five methods, respectively (Figure 3). From the above studies, mrMLM, FASTmrMLM, and ISIS EM-BLASSO detected a larger number of QTNs among the five methods generally (Figure 4A). In different arrangements of two methods, the number of common QTNs detected by mrMLM combined with FASTmrMLM was large (Figure 4B).

### Distribution of Superior Alleles in the FW-RILs

**FIGURE 5:**
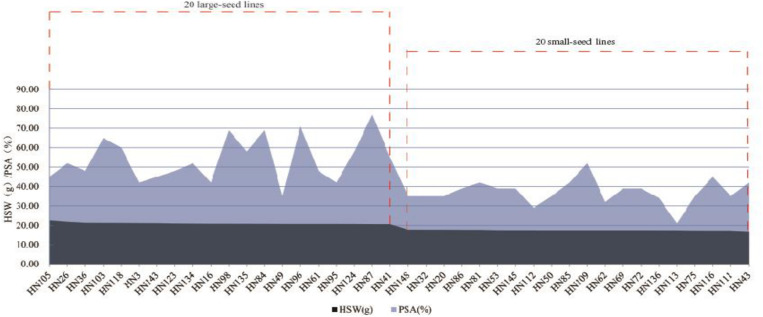
Distribution of superior allele percentages and the hundred-seed weight in the 40 smaller and larger-seed lines.

We identified 20 large-seed lines with high HSW and 20 small-seed lines with low HSW based on the average phenotypic values of 144 FW-RILs in 20 environments (Table 4). The HSW phenotypic values of the 20 large-seed lines ranged from 20.65 to 22.55 g. For each large-seed line, the percentage of superior alleles (PSAs) for the 31 common QTNs ranged from 35 to 77%; nine lines (45%) showed a PSA of less than 50%. The phenotypic values for the 20 small-seed lines ranged from 16.53 to 17.79 g, and the PSA ranged from 21 to 52% (Table 4). Therefore, the large-seed lines had a higher PSA than the small-seed lines (Figure 5).

**TABLE 4:** Phenotypic averages of hundred-seed weight and proportion of superior alleles in 40 lines across 31 common QTNs.

**Line**	**HSW (g)**	**PSA (%)**	**Line**	**HSW (g)**	**PSA (%)**
HN105	22.55	45	**HN148**	**17.79**	**35**
HN26	21.82	52	**HN32**	**17.78**	**35**
HN36	21.30	48	**HN20**	**17.77**	**35**
HN103	21.25	65	**HN86**	**17.69**	**39**
HN118	21.22	60	**HN81**	**17.68**	**42**
HN3	21.14	42	**HN53**	**17.52**	**39**
HN143	21.12	45	**HN145**	**17.51**	**39**
HN123	20.97	48	**HN112**	**17.50**	**29**
HN134	20.89	52	**HN50**	**17.44**	**35**
HN16	20.83	42	**HN85**	**17.43**	**42**
HN98	20.82	69	**HN109**	**17.29**	**52**
HN135	20.80	58	**HN62**	**17.24**	**32**
HN84	20.79	69	**HN69**	**17.19**	**39**
HN49	20.75	35	**HN72**	**17.15**	**39**
HN96	20.74	71	**HN136**	**17.11**	**34**
HN61	20.74	48	**HN113**	**17.08**	**21**
HN95	20.73	42	**HN75**	**16.95**	**35**
HN124	20.71	58	**HN116**	**16.93**	**45**
HN87	20.66	77	**HN111**	**16.92**	**35**
HN41	20.65	55	**HN43**	**16.53**	**42**

*Bold font indicates the 20 lines with smaller seeds, and non-bold font indicates the 20 lines with larger seeds.*

**FIGURE 6:**
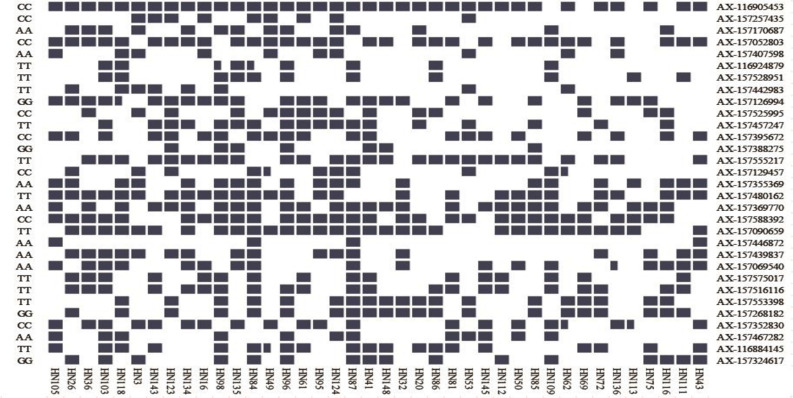
Heat map of the superior allele distribution for the 31 common QTNs in the 40 smaller and larger-seed lines. Blue and white colors represent superior and inferior alleles, respectively.

The PSA range for each common QTN in the 20 large-seed lines was 15–100%, with 18 QTNs having PSAs of ≥50%. Among the 20 small-seed lines, PSAs ranged from 5 to 80%, and nine QTNs had PSAs of >50% (Table 5 and Figure 6). The number of common QTNs with PSAs ≥50% in 20 large-seed lines was higher than 20 small-seed lines. Based on these results, we can find elite lines by identifying the superior allele ratio of high-yield soybeans.

**TABLE 5:** Superior alleles and their proportions of 31 common QTNs in 20 larger seed lines and 20 smaller seed lines.

**QTN**	**Superior allele**	**PSA (%)^a^**	**PSA (%)^b^**	**QTN**	**Superior allele**	**PSA (%)^a^**	**PSA (%)^b^**
AX-116905453	CC	100	80	AX-157480162	TT	80	55
AX-157257435	CC	40	5	AX-157369770	AA	70	65
AX-157170687	AA	55	15	AX-157588392	CC	80	70
AX-157052803	CC	85	70	AX-157090659	TT	85	70
AX-157407598	AA	35	15	AX-157446872	AA	15	5
AX-116924879	TT	35	10	AX-157439837	AA	60	25
AX-157528951	TT	35	20	AX-157069540	AA	45	43
AX-157442983	TT	30	10	AX-157575017	TT	50	30
AX-157126994	GG	78	45	AX-157516116	TT	60	45
AX-157525995	CC	50	25	AX-157553398	TT	40	55
AX-157457247	TT	65	25	AX-157268182	GG	45	60
AX-157395672	CC	65	40	AX-157352830	CC	60	35
AX-157388275	GG	25	10	AX-157467282	AA	35	25
AX-157555217	TT	70	65	AX-116884145	TT	53	45
AX-157129457	CC	38	13	AX-157324617	GG	40	40
AX-157355369	AA	60	40	–	–	–	–

*^a^Indicates the percentage of superior allele of each common QTN in 20 large-seed lines. ^b^Indicates the percentage of superior allele of each common QTN in 20 small-seed lines.*

We identified several superior alleles that were present in various large-seed lines; *AX-116905453, AX-157457247, AX-157588392*, and *AX-157090659* were all present in HN96, HN61, HN95, HN124, HN87, and HN41 (Figure 6). The superior allele AX-116905453 was present in all 20 large-seed lines. We believe that superior alleles may have an important effect on the HSW of soybeans. In further research, we hope to use this information to develop larger seed size soybean varieties through marker-assisted selection.

### Potential Candidate Genes Determined Based on Common QTNs

**FIGURE 7:**
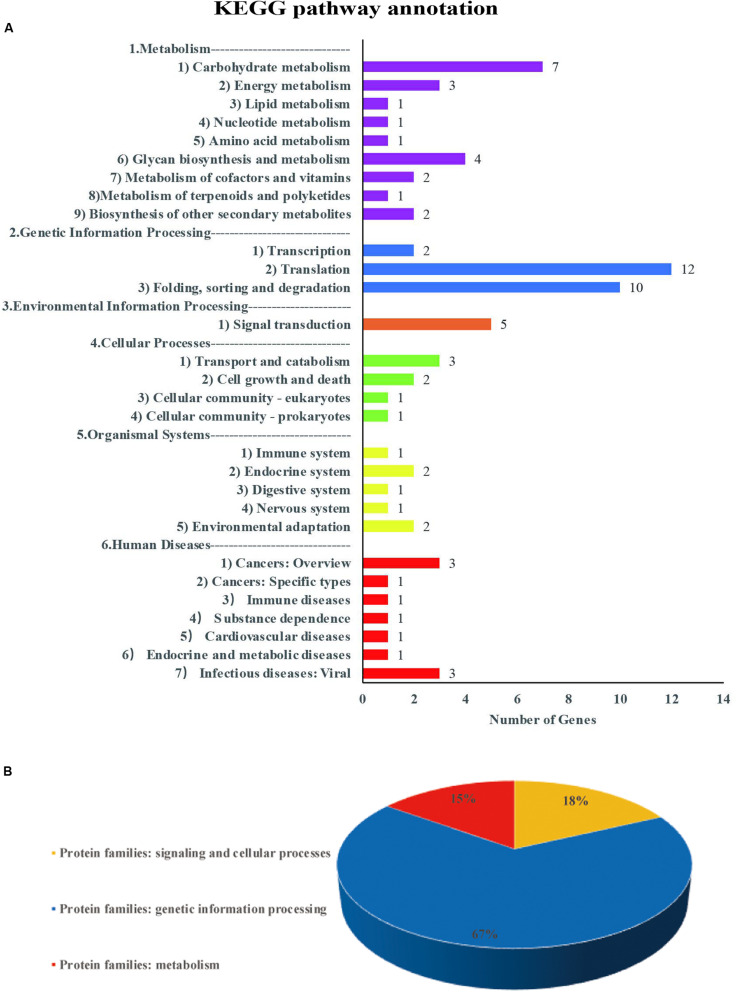
Information on pathways and orthologous protein families of 51 genes. Panel **(A)** shows the information on pathway. Panel **(B)** shows the information on orthologous protein families.

For each common QTN, we use the LD decay distance as the interval range to find candidate genes. Because the population we selected was not a natural group, but an FW-RIL population, the LD decay distance was very large. We therefore chose the range of potential candidate genes according to the location with the largest decay rate. As the LD decayed fastest before 200 kb and then tended to flatten (Figure 2A), we searched for potential candidate genes at 100-kb intervals on either side of each QTN. We identified 635 genes in this interval, of which 129 were highly expressed during seed formation (Supplementary Table S7). Based on the annotation data, 51 of the 129 genes were annotated in 29 pathways and three protein families in the KEGG database (Supplementary Tables S5, S6 and Figure 7). Three of these are potential candidate genes based on their annotation information and function in metabolic pathways (Table 6).

**TABLE 6:** Details of three candidate genes annotated in the KEGG database.

**QTN name**	**Gene name^a^**	**Chr**	**Position (bp)**	**KO number**	**Annotation**
AX-157525995	*Glyma.06G321900*	6	51040796–51041580	K08905	psaG; photosystem I subunit V
AX-157555217	*Glyma.08G365900*	8	47673091–47677260	K01809	manA, MPI; mannose-6-phosphate isomerase [EC:5.3.1.8]
AX-116884145	*Glyma.20G240000*	20	47137688–47144012	K12373	HEXA_B; hexosaminidase [EC:3.2.1.52]

*^*a*^Indicates the gene which correlates with the QTN.*

## Discussion

Most genetic analyses of yield-related traits in soybean, such as HSW, have been based on RILs arising from the hybridization of two parents used for QTL mapping analyses. Sites detected in these analyses will therefore be suitable only for that specific population. Most mapping populations involve a limited number of recombination, and few molecular markers can be used; fine mapping is challenging. Four-way RILs were derived from four parents, and there may be one to four alleles in a QTL. As long as there is a significant difference in genetic effect between the two alleles in a QTL, this QTL is detected. Therefore, the intensity of QTL detection and superior allele selection are improved. In recent years, with the rapid development of GWAS, natural population is generally used as research materials to identify HSW QTL in soybean. The FW-RILs used in this study are the MAGIC (multiparent advanced generation intercross) lines, which were artificially configured with good linkage relationship, whereas the natural population has no linkage relationship. The number of parents of the FW-RILs is limited, and the population structure is not obvious compared with the natural population composed of many germplasm resources. Thus, the false-positive rate of FW-RILs is lower than that of the natural population; the statistical power and QTL positioning accuracy are higher.

Previous studies have shown that HSW is a typical quantitative trait controlled by multiple genes, and it is controlled by many microgene loci under different genetic conditions. A GWAS using 31,283 SNPs and CMLM detected five loci associated with HSW (Copley et al., 2018). Yan et al. (2017) used SoySNP50K BeadChip to genotype more than 42,000 SNPs, and the MLM accounting for both population structure and kinship was conducted for GWAS. Eight SNPs located on chromosomes 4 and 17 were significantly associated with HSW. The variation explained by significant markers (*R*^2^) ranged from 6.9 to 13.2%. Contreras-Sota et al. (2017) used SNP markers and MLM considering Q + K models for GWAS to identify genomic regions controlling HSW. Seven SNPs were significantly associated with HSW on chromosomes 5, 7, 11, and 12 across the locations under study, and *R*^2^ ranged from 13.2 to 31.2%. Although high-density SNP markers were applied in these experiments, statistical methods (Yu et al., 2006) treating single loci mainly detect QTLs with high heritability and neglect those with moderate or low heritability. The five multilocus GWAS methods used in this study greatly reduce the false positives in the result and increase the credibility of the results. All potential QTNs with large or small effects in different statistical models could be detected by the methods.

We performed a multilocus genome-wide association analysis of HSW in soybean using a high-density SNP chip and mapped 31 common QTNs in 20 environments (Table 3). Currently, the SoyBase database includes 314 targeted HSW QTLs^1^. The 11 common QTNs identified in this study were present in the SoyBase database: *AX-157439837*, *AX-157129457*, *AX-157090659*, *AX-157467282*, *AX-157324617*, *AX-157407598*, *AX-157369770*, *AX-157528951*, *AX-157388275*, *AX-157442983*, and *AX-157257435*. Quantitative trait loci physically close to these were identified previously (Mian et al., 1996; Hyten et al., 2004; Chen et al., 2007; Li D. et al., 2008; Teng et al., 2009; Liu et al., 2011; Han et al., 2012; Kato et al., 2014; Gai et al., 2016; Hacisalihoglu et al., 2018). This supports the accuracy of our study and validates the authenticity of the loci involved in seed size formation.

Based on the common QTNs detected in this study and the annotated pathways associated with these QTNs, we identified three genes that may be associated with HSW in soybean (Table 6). *Glyma.06G321900* is associated with psaG, an intrinsic membrane protein associated with Photosystem I (PSI). *psaG* is a nuclear-encoded gene corresponding to PSI subunit V of spinach (Steppuhn et al., 1988). Further experiments evaluate the potential role of subunit V in PSI function under water-deficit stress (Wood and Duff, 1999). Photosynthesis converts light energy into chemical energy for plant growth, increasing crop yield. Therefore, we speculate that this gene increases photosynthesis in the absence of water, which indirectly affects the HSW of soybean. *Glyma.08G365900* is related to mannose phosphate isomerase (PMI), and the PMI (*manA*) gene encodes a mannose-6-phosphate transferase, catalyzing the conversion of 6-phosphoric acid mannose to fructose 6-phosphate. Cells can metabolize fructose via the glycolytic pathway (Reed et al., 2001), rendering mannose a carbon source for the growth of transformed cells, and thus the plant exhibits a more prominent growth advantage. We speculate that PMI (*manA*) indirectly controls the HSW of soybean plants. *Glyma.20G240000* is associated with hexosaminidase (Hex). During fruit ripening, β-Hex releases a large amount of free *N*-glycans from glycoprotein polypeptide chains (Yunovitz and Gross, 1994). Free *N*-glycans are important signaling molecules that promote fruit ripening (Handa et al., 1985; Priem and Gross, 1992), possibly through β-Hex triggering ethylene synthesis systems (Jagadeesh et al., 2004). We therefore suggest that Hex controls soybean seed development by affecting the biosynthesis of phytohormones.

### Summary

We identified 31 QTNs using a five-multilocus GWAS method. An analysis of the common QTNs identified three candidate genes. By analyzing the ratio of superior alleles in large- and small-seed lines, we established that the superior alleles of these common QTNs might be useful for molecular marker-assisted breeding of soybean plants with larger seeds.

## Data Availability Statement

The genotyping data is available at https://figshare.com/s/84eb97ad5c5e523072e5.

## Author Contributions

WL and HN conceived and designed the experiments. JS, XT, YW, YF, XL, JW, CY, SJ, and XS performed the field experiments. SL and ZT performed the genome sequencing. ZQ, KZ, and HN analyzed and interpreted the results. ZQ and HN drafted the manuscript and all authors contributed to manuscript revision. WL acquired the funding. All authors contributed to the article and approved the submitted version.

## Conflict of Interest

The authors declare that the research was conducted in the absence of any commercial or financial relationships that could be construed as a potential conflict of interest.

## Footnotes

1 http://www.soybase.org/
