# Supplementary material for: Identification of QTNs Controlling 100-Seed Weight in Soybean Using Multilocus Genome-Wide Association Studies
Source: Front Genet. 2020 Jul 16;11:689. doi: 10.3389/fgene.2020.00689 (PMC7378803; doi:10.3389/fgene.2020.00689)
Supplement: Supplementary file 1 [file Data_Sheet_1.pdf]

Table S1 | Summary of planting conditions of 20 environments

Environment	Year	Locations	Experimental treatment		
			Planting Density (× 10^5 plant/hm^2)	Fertilizer (N / P2O5/ K2O) (kg/hm^2)	Sowing Date
E1	2013	Keshan	28	18/46/30	15/May
E2	2015	Harbin	28	18/46/30	10/May
E3	2015	Harbin	32	18/46/30	10/May
E4	2014	Keshan	30	18/46/30	10/May
E5	2014	Harbin	22	18/46/30	10/May
E6	2014	Harbin	22	18/46/30	25/May
E7	2016	Acheng	22	18/46/30	15/May
E8	2016	Acheng	22	18/46/30	05/May
E9	2016	Acheng	25	18/46/30	05/May
E10	2016	Acheng	22	36/92/60	05/May
E11	2016	Shuangcheng	22	18/46/30	15/May
E12	2016	Shuangcheng	22	18/46/30	10/May
E13	2016	Shuangcheng	25	18/46/30	10/May
E14	2016	Shuangcheng	22	36/92/60	10/May
E15	2016	Harbin	22	18/46/30	17/May
E16	2016	Harbin	22	18/46/30	07/May
E17	2016	Harbin	25	18/46/30	07/May
E18	2016	Harbin	22	36/92/60	07/May
E19	2017	Shuangcheng	22	18/46/30	10/May
E20	2018	Acheng	22	18/46/30	10/May

Table S2｜Average phenotypic data（unit: g）of hundred-seed weight of four parents in 20 environments

	Kenfeng14	Kenfeng15	Kenfeng19	Heinong48
E1	20.57	19.50	16.55	21.95
E2	NA	NA	NA	NA
E3	NA	NA	NA	NA
E4	20.46	21.94	20.84	21.44
E5	10.67	17.88	18.17	21.14
E6	23.69	19.16	17.78	23.73
E7	NA	NA	NA	NA
E8	18.28	17.24	11.07	22.63
E9	NA	NA	NA	NA
E10	NA	NA	NA	NA
E11	NA	NA	NA	NA
E12	19.36	21.57	20.29	21.60
E13	NA	NA	NA	NA
E14	NA	NA	NA	NA
E15	NA	NA	NA	NA
E16	18.00	17.28	19.07	19.28
E17	NA	NA	NA	NA
E18	NA	NA	NA	NA
E19	18.89	17.94	23.16	19.63
E20	22.20	22.40	24.48	18.52

Table S3 | Descriptive statistics of 144 FW-RILs HSW (unit: g) in 20 environments

	Average	Standard error	Median	Mode	Standard deviation	Variance	Kurtosis	Skewness	Minimum	Maximum
E1	18.75	0.17	18.74	17.35	2.06	4.25	1.68	-0.25	11.07	25.18
E2	19.74	0.32	20.05	16.30	3.62	13.07	0.25	-0.01	10.11	30.74
E3	19.54	0.31	19.87	13.87	3.57	12.76	0.22	-0.07	9.65	30.39
E4	19.24	0.21	19.01	17.40	2.35	5.53	0.87	0.20	13.01	26.4
E5	19.23	0.21	19.00	17.40	2.36	5.56	0.86	0.21	13.01	26.4
E6	20.42	0.19	19.91	18.66	2.21	4.87	-0.16	0.41	14.77	25.73
E7	18.55	0.21	18.75	19.32	2.32	5.41	-0.12	0.18	13.48	25.25
E8	17.81	0.21	17.82	17.82	2.26	5.10	-0.09	-0.31	11.07	22.63
E9	16.64	0.25	16.47	14.37	2.27	5.17	-0.50	0.29	12.53	22.69
E10	16.71	0.19	16.54	14.65	1.94	3.77	0.34	0.31	12.46	23.41
E11	17.67	0.20	17.72	17.04	2.24	5.03	0.15	0.44	12.92	24.85
E12	19.26	0.18	19.27	19.27	2.09	4.36	-0.35	-0.02	14.56	24.56
E13	19.53	0.20	19.58	19.79	2.34	5.49	0.37	0.23	14.10	26.68
E14	19.97	0.18	19.74	17.20	2.12	4.50	-0.56	0.24	15.95	25.29
E15	18.18	0.19	18.14	18.54	2.25	5.06	-0.32	0.01	12.45	23.33
E16	18.73	0.21	18.51	17.00	2.41	5.79	-0.20	0.24	12.96	25.47
E17	19.04	0.27	18.32	17.12	3.09	9.57	0.28	0.68	13.21	28.60
E18	18.94	0.21	18.58	15.44	2.44	5.96	-0.61	0.48	15.06	24.75
E19	20.88	0.21	21.23	22.10	2.53	6.40	-0.36	-0.25	14.46	27.02
E20	22.45	0.26	23.17	23.47	3.07	9.44	0.03	-0.33	14.64	31.05

Table S4 | 118 significant QTNs detected in 20 environments

Environment	Method	QTN name	Chro	Marker Position (bp)	QTN effect	LOD score	r^2 (%)	Genotype for code 1
E1	pLARmEB	AX-157191877	10	41948285	-0.35	3.26	2.76	TT
E1	pLARmEB	AX-157480202	19	44840212	-0.69	4.82	5.61	CC
E1	FASTmrMLM	AX-157575017	16	32946305	-0.43	3.06	4.35	GG
E1	ISIS EM-BLASSO	AX-157575017	16	32946305	-0.45	3.24	4.78	GG
E2	FASTmrMLM	AX-157369770	11	32617288	-0.87	3.80	5.02	GG
E2	ISIS EM-BLASSO	AX-157378148	11	32879122	-0.82	3.23	4.90	CC
E2	FASTmrMLM	AX-157395672	7	37249194	-0.77	3.67	4.49	TT
E2	ISIS EM-BLASSO	AX-157395672	7	37249194	-0.82	4.26	5.13	TT
E2	FASTmrMLM	AX-157525995	6	51088854	0.92	4.48	6.43	CC
E2	ISIS EM-BLASSO	AX-157525995	6	51088854	0.66	3.08	3.28	CC
E2	FASTmrMLM	AX-157549780	19	36241392	-1.11	3.38	4.70	TT
E2	ISIS EM-BLASSO	AX-157578963	4	550710	0.94	4.74	6.35	AA
E2	FASTmrMLM	AX-157588392	12	17219655	-1.01	4.20	6.64	TT
E3	ISIS EM-BLASSO	AX-157277972	2	47531205	0.68	3.04	3.61	AA
E3	ISIS EM-BLASSO	AX-157369770	11	32617288	-0.75	3.07	3.78	GG
E3	ISIS EM-BLASSO	AX-157525995	6	51088854	0.74	3.90	4.24	CC
E3	ISIS EM-BLASSO	AX-157588392	12	17219655	-0.97	4.26	6.36	TT
E5	ISIS EM-BLASSO	AX-157099829	17	37818306	0.54	3.46	5.18	AA
E5	mrMLM	AX-157301945	19	1272744	-0.67	3.04	8.09	T
E5	mrMLM	AX-157447975	10	45306473	-1.13	3.48	11.10	G
E6	mrMLM	AX-116924879	3	5093950	-0.82	3.16	11.33	C
E6	FASTmrMLM	AX-116924879	3	5093950	-0.60	3.70	6.23	CC
E6	pLARmEB	AX-116924879	3	5093950	-0.60	4.13	6.23	CC
E6	ISIS EM-BLASSO	AX-116924879	3	5093950	-0.73	4.33	8.96	CC

E6	pLARmEB	AX-157093832	6	8528841	0.00	3.15	0.00	AA
E6	pLARmEB	AX-157496214	13	35413703	0.00	3.10	0.00	GG
E6	mrMLM	AX-157516116	16	33525379	-0.72	3.35	10.30	A
E6	FASTmrMLM	AX-157516116	16	33525379	-0.55	4.44	6.08	AA
E6	pLARmEB	AX-157516116	16	33525379	-0.55	5.65	6.09	AA
E7	pLARmEB	AX-157161830	16	13196270	-0.55	4.30	5.00	AA
E7	FASTmrMLM	AX-157324617	20	42347884	0.54	3.31	5.35	GG
E7	ISIS EM-BLASSO	AX-157324617	20	42347884	0.66	3.73	7.38	GG
E7	ISIS EM-BLASSO	AX-157361226	10	3035246	0.84	5.82	9.79	AA
E7	pLARmEB	AX-157392584	8	7110621	0.85	4.36	8.07	TT
E7	ISIS EM-BLASSO	AX-157499177	18	14707082	-0.57	3.94	5.56	CC
E7	FASTmrMLM	AX-157528951	3	6197954	-0.66	3.51	5.84	CC
E7	ISIS EM-BLASSO	AX-157528951	3	6197954	-0.94	6.68	11.04	CC
E9	FASTmrEMMA	AX-157257435	2	43283682	2.05	3.32	12.83	CC
E9	ISIS EM-BLASSO	AX-157257435	2	43283682	0.70	3.22	5.91	CC
E9	ISIS EM-BLASSO	AX-157392622	15	18061095	-0.62	3.60	6.19	TT
E9	mrMLM	AX-157555217	8	47740465	0.95	3.85	13.46	T
E9	FASTmrMLM	AX-157555217	8	47740465	0.87	4.22	11.42	TT
E9	ISIS EM-BLASSO	AX-157557627	16	2907490	0.89	4.78	11.57	TT
E10	FASTmrMLM	AX-116956385	10	39661625	0.00	3.11	0.00	TT
E10	mrMLM	AX-157170687	3	1393672	0.72	3.08	12.40	A
E10	FASTmrMLM	AX-157170687	3	1393672	0.56	3.98	7.52	AA
E10	ISIS EM-BLASSO	AX-157170687	3	1393672	0.54	3.30	6.94	AA
E10	FASTmrMLM	AX-157439837	15	12672673	1.16E-05,	3.60	3.53E-09	AA
E10	FASTmrEMMA	AX-157439837	15	12672673	1.01	3.45	6.34	AA
E10	pLARmEB	AX-157439837	15	12672673	0.46	3.22	5.15	AA

E10	mrMLM	AX-157442983	4	6484678	1.15	4.01	12.52	T
E10	FASTmrMLM	AX-157442983	4	6484678	0.88	3.02	7.33	TT
E10	FASTmrEMMA	AX-157442983	4	6484678	1.98	3.38	10.44	TT
E10	pLARmEB	AX-157442983	4	6484678	0.95	3.56	8.03	TT
E10	ISIS EM-BLASSO	AX-157442983	4	6484678	0.92	3.77	7.98	TT
E11	FASTmrEMMA	AX-116906756	9	37756566	-1.10	3.10	5.61	AA
E12	mrMLM	AX-157069540	16	37223445	-0.67	4.17	10.06	G
E12	FASTmrMLM	AX-157069540	16	37223445	-0.55	4.17	6.91	GG
E12	ISIS EM-BLASSO	AX-157133176	6	7513077	0.42	3.51	3.84	GG
E12	mrMLM	AX-157388275	8	46069989	-0.88	4.08	11.96	A
E12	FASTmrMLM	AX-157388275	8	46069989	-0.70	4.08	7.74	AA
E12	ISIS EM-BLASSO	AX-157562151	16	37417515	-0.60	5.59	8.20	AA
E13	ISIS EM-BLASSO	AX-157062854	10	27849788	-0.80	3.39	7.87	CC
E14	mrMLM	AX-157058012	3	362748	-0.62	3.70	7.89	G
E14	mrMLM	AX-157069642	5	37699648	0.70	4.62	9.50	C
E14	mrMLM	AX-157457247	7	3254514	-0.71	4.81	10.00	C
E14	FASTmrMLM	AX-157457247	7	3254514	-0.49	3.23	5.06	CC
E14	ISIS EM-BLASSO	AX-157457247	7	3254514	-0.52	3.66	5.72	CC
E14	mrMLM	AX-157467282	20	43174088	-0.77	4.71	11.61	G
E14	FASTmrMLM	AX-157467282	20	43174088	-0.43	3.67	3.93	GG
E14	mrMLM	AX-157577636	15	2837402	0.66	3.16	6.38	G
E15	mrMLM	AX-157052803	3	1722938	0.99	4.38	12.96	C
E15	pLARmEB	AX-157052803	3	1722938	0.90	4.95	10.80	CC
E15	ISIS EM-BLASSO	AX-157052803	3	1722938	0.86	3.86	10.08	CC
E15	FASTmrMLM	AX-157553398	18	56507541	0.48	3.52	4.43	TT
E15	pLARmEB	AX-157553398	18	56507541	0.49	3.05	4.63	TT

E16	mrMLM	AX-157126994	6	49959758	-0.99	5.67	14.03	A
E16	FASTmrMLM	AX-157126994	6	49959758	-0.83	5.67	10.74	AA
E16	pLARmEB	AX-157126994	6	49959758	-0.78	5.31	9.52	AA
E16	ISIS EM-BLASSO	AX-157126994	6	49959758	-0.64	3.87	6.35	AA
E16	mrMLM	AX-157268182	18	56589017	-0.81	3.89	9.95	A
E16	FASTmrMLM	AX-157268182	18	56589017	-0.60	3.89	6.05	AA
E16	pLARmEB	AX-157268182	18	56589017	-0.54	3.48	4.92	AA
E16	mrMLM	AX-157407598	3	39988963	0.90	3.52	9.03	A
E16	FASTmrMLM	AX-157407598	3	39988963	0.61	3.52	4.59	AA
E17	pLARmEB	AX-117470527	7	28002677	0.58	3.03	3.48	AA
E17	FASTmrMLM	AX-157267394	12	15249724	-0.69	3.14	4.09	CC
E17	pLARmEB	AX-157304673	10	43619953	-0.59	3.29	2.64	AA
E17	FASTmrMLM	AX-157406932	18	56653034	-0.84	3.96	6.20	GG
E17	ISIS EM-BLASSO	AX-157453982	11	15172874	0.85	4.07	7.47	CC
E17	FASTmrMLM	AX-157501565	17	7716976	0.86	4.05	6.51	GG
E18	mrMLM	AX-116905453	2	44205396	1.43	5.43	15.07	C
E18	FASTmrMLM	AX-116905453	2	44205396	1.23	5.43	12.14	CC
E18	FASTmrEMMA	AX-116905453	2	44205396	2.23	4.64	8.42	CC
E18	pLARmEB	AX-116905453	2	44205396	1.13	5.26	10.12	CC
E18	ISIS EM-BLASSO	AX-116905453	2	44205396	1.15	5.48	10.74	CC
E18	pLARmEB	AX-157100184	9	3227391	0.48	3.80	3.82	AA
E18	pLARmEB	AX-157129457	9	40507825	0.59	3.71	3.80	CC
E18	ISIS EM-BLASSO	AX-157129457	9	40507825	0.59	3.13	3.82	CC
E18	mrMLM	AX-157355369	10	49273669	0.90	3.52	12.43	A
E18	FASTmrMLM	AX-157355369	10	49273669	0.63	3.52	6.50	AA
E18	FASTmrEMMA	AX-157355369	10	49273669	1.25	3.70	6.00	AA

E18	pLARmEB	AX-157355369	10	49273669	0.70	5.02	8.03	AA
E18	ISIS EM-BLASSO	AX-157355369	10	49273669	0.56	3.00	5.19	AA
E18	ISIS EM-BLASSO	AX-157459580	9	2897319	0.48	3.44	3.74	AA
E19	mrMLM	AX-116884145	20	47162277	-0.75	3.19	8.45	C
E19	FASTmrMLM	AX-116884145	20	47162277	-0.50	3.01	3.94	CC
E19	ISIS EM-BLASSO	AX-117062897	13	31645253	0.72	3.33	5.07	AA
E19	mrMLM	AX-157352830	18	56836743	0.89	3.75	11.44	C
E19	FASTmrMLM	AX-157352830	18	56836743	0.71	4.55	7.76	CC
E19	FASTmrMLM	AX-157429431	17	30410095	0.00	3.02	0.00	AA
E19	FASTmrEMMA	AX-157443237	6	15621215	0.00	4.64	0.00	GG
E19	mrMLM	AX-157480162	10	50172467	0.85	3.14	8.82	T
E19	FASTmrMLM	AX-157480162	10	50172467	0.60	3.12	4.66	TT
E20	FASTmrEMMA	AX-157090659	14	881999	1.57	3.28	4.80	TT
E20	ISIS EM-BLASSO	AX-157090659	14	881999	0.80	3.01	5.21	TT
E20	FASTmrEMMA	AX-157446872	14	37252443	-1.75	3.86	6.61	GG
E20	ISIS EM-BLASSO	AX-157446872	14	37252443	-0.88	4.15	7.80	GG

Table S5丨The information of pathways and protein families of 51 genes annotated in the KEGG.

QTN name	Gene namea	Chr	Position(bp)	KO number	KEGG pathway	protein family
AX-116905453	Glyma.02G254500	2	44151303..44155647	K12947	Genetic Information Processing:Folding, sorting and degradation	
AX-116905453	Glyma.02G255000	2	44207467..44209844	K09872		signaling and cellular processes
AX-116905453	Glyma.02G255500	2	44240358..44242714	K02941	Genetic Information Processing:Translation	genetic information processing
AX-157257435	Glyma.02G244000	2	43200089..43204490	K01915	Metabolism:Carbohydrate metabolism;Energy metabolism;Amino acid metabolism.Cellular Processes:Cell growth and death.Environmental Information Processing:Signal transduction.Organismal Systems:Nervous system	signaling and cellular processes
AX-157257435	Glyma.02G244100	2	43222701..43224588	K07766		
AX-157170687	Glyma.03G013200	3	1337139..1343939	K12614	Genetic Information Processing:Folding, sorting and degradation	genetic information processing
AX-157170687	Glyma.03G013600	3	1379993..1390392	K15440		genetic information processing
AX-157170687	Glyma.03G014300	3	1427595..1433362	K15095	Metabolism:Metabolism of terpenoids and polyketides	
AX-157407598	Glyma.03G188500	3	39957460..39962189	K13126	Genetic Information Processing:Translation;Folding, sorting and degradation	genetic information processing
AX-157407598	Glyma.03G189300	3	40021744..40024023	K02896	Genetic Information Processing:Translation	genetic information processing
AX-157407598	Glyma.03G189700	3	40049277..40056481	K00873	Organismal Systems:Endocrine system.Human Diseases:Cancers: Overview;Endocrine and metabolic diseases;Infectious diseases: Viral.Metabolism:Carbohydrate metabolism;Nucleotide metabolism	signaling and cellular processes;genetic information processing

AX-157442983	Glyma.04G076700	4	6445919..6452821	K13201		genetic information processing
AX-157442983	Glyma.04G077400	4	6499898..6503759	K02738	Genetic Information Processing:Folding, sorting and degradation	genetic information processing; metabolism
AX-157442983	Glyma.04G078200	4	6541151..6546684	K03036	Genetic Information Processing:Folding, sorting and degradation.Human Diseases:Infectious diseases: Viral	genetic information processing
AX-157126994	Glyma.06G310200	6	49870537..49873394	K13280	Genetic Information Processing:Folding, sorting and degradation	metabolism
AX-157126994	Glyma.06G310400	6	49880338..49884322	K11271		genetic information processing
AX-157525995	Glyma.06G321900	6	51040796..51041580	K08905	Metabolism:Energy metabolism	metabolism;
AX-157525995	Glyma.06G322300	6	51058001..51065788	K14945		genetic information processing
AX-157525995	Glyma.06G323000	6	51103666..51106094	K02868	Genetic Information Processing:Translation	genetic information processing
AX-157395672	Glyma.07G202300	7	37166660..37169027	K13257	Metabolism:Biosynthesis of other secondary metabolites	metabolism
AX-157395672	Glyma.07G202500	7	37177013..37177959	K11253	Human Diseases:Cancers: Overview;Immune diseases;Substance dependence	signaling and cellular processes;genetic information processing
AX-157395672	Glyma.07G204400	7	37336384..37340546	K00721	Metabolism:Glycan biosynthesis and metabolism	metabolism
AX-157388275	Glyma.08G345200	8	46015608..46023940	K06126	Metabolism:Metabolism of cofactors and vitamins	
AX-157388275	Glyma.08G346000	8	46083151..46086614	K03109	Genetic Information Processing:Folding, sorting and degradation	signaling and cellular processes
AX-157388275	Glyma.08G346500	8	46138146..46140731	K02995	Genetic Information Processing:Translation	genetic information processing
AX-157555217	Glyma.08G365900	8	47673091..47677260	K01809	Metabolism:Carbohydrate metabolism.	
AX-157555217	Glyma.08G367400	8	47783600..47785134	K17781		signaling and cellular processes;genetic information processing
AX-157129457	Glyma.09G179500	9	40420404..40427085	K14376	Genetic Information Processing:Translation	genetic information processing
AX-157480162	Glyma.10G279900	10	50130564..50136128	K02729	Genetic Information Processing:Folding, sorting and	genetic information processing;metabolism

					degradation	
AX-157369770	Glyma.11G231000	11	32666235..32668355	K02865	Genetic Information Processing:Translation	genetic information processing
AX-157090659	Glyma.14G010200	14	788305..792206	K12872	Genetic Information Processing:Transcription	genetic information processing
AX-157090659	Glyma.14G010400	14	802172..805618	K02259	Environmental Information Processing:Signal transduction.Organismal Systems:Environmental adaptation.Metabolism:Energy metabolism;Metabolism of cofactors and vitamins	genetic information processing
AX-157090659	Glyma.14G011600	14	887156..890657	K04079	Genetic Information Processing:Folding, sorting and degradation.Environmental Information Processing:Signal transduction.Cellular Processes:Cell growth and death.Organismal Systems:Endocrine system;Immune system;Environmental adaptation.Human Diseases:Cancers: Overview;Cancers: Specific types;Cardiovascular diseases	signaling and cellular processes;genetic information processing;metabolism
AX-157439837	Glyma.15G152000	15	12592655..12594969	K09286		genetic information processing
AX-157439837	Glyma.15G153300	15	12725123..12726992	K15032		genetic information processing
AX-157439837	Glyma.15G153400	15	12730592..12733439	K15032		genetic information processing
AX-157069540	Glyma.16G213300	16	37138811..37144846	K07151	Metabolism:Glycan biosynthesis and metabolism.Genetic Information Processing:Folding, sorting and degradation	metabolism
AX-157069540	Glyma.16G215500	16	37284766..37290393	K03245	Genetic Information Processing:Translation	genetic information processing
AX-157553398	Glyma.18G284200	18	56482736..56486831	K03247	Genetic Information Processing:Translation.Human Diseases:Infectious diseases: Viral.	signaling and cellular processes;genetic information processing
AX-157553398	Glyma.18G284300	18	56489793..56496952	K01634	Metabolism:Lipid metabolism.Environmental Information Processing:Signal transduction.	

AX-157268182	Glyma.18G286200	18	56637471..56646826	K12373	Metabolism:Carbohydrate metabolism;Glycan biosynthesis and metabolism.Cellular Processes:Transport and catabolism	genetic information processing
AX-157268182	Glyma.18G286400	18	56665160..56667323	K02893	Genetic Information Processing:Translation	genetic information processing
AX-157352830	Glyma.18G287400	18	56740754..56748259	K00164	Metabolism:Carbohydrate metabolism	
AX-157352830	Glyma.18G287600	18	56754676..56759595	K07901	Environmental Information Processing:Signal transduction.Cellular Processes:Transport and catabolism;Cellular community - eukaryotes.Organismal Systems:Digestive system	signaling and cellular processes;genetic information processing
AX-157467282	Glyma.20G193100	20	43207414..43208918	K02933	Genetic Information Processing:Translation	genetic information processing
AX-116884145	Glyma.20G240000	20	47137688..47144012	K12373	Metabolism:Carbohydrate metabolism;Glycan biosynthesis and metabolism.Cellular Processes:Transport and catabolism	genetic information processing
AX-116884145	Glyma.20G240500	20	47172307..47177872	K12830	Genetic Information Processing:Transcription	genetic information processing
AX-116884145	Glyma.20G240700	20	47188810..47190879	K02918	Genetic Information Processing:Translation	genetic information processing
AX-116884145	Glyma.20G240800	20	47192875..47195439	K01728	Metabolism:Carbohydrate metabolism.Cellular Processes:Cellular community - prokaryotes	
AX-157324617	Glyma.20G184400	20	42259262..42264549	K12736		genetic information processing
AX-157324617	Glyma.20G185700	20	42428886..42431148	K13081	Metabolism:Biosynthesis of other secondary metabolites	

TABLES6 | Details of 51 genes annotated in the KEGG database.

QTN name	Gene namea	Chr	Position	KO number	Annotation
AX-116905453	Glyma.02G254500	2	44151303..44155647	K12947	SPCS2, SPC2;signal peptidase complex subunit 2 [EC:3.4.-.-]
AX-116905453	Glyma.02G255000	2	44207467..44209844	K09872	PIP;aquaporin PIP
AX-116905453	Glyma.02G255500	2	44240358..44242714	K02941	RPLP0;large subunit ribosomal protein LP0
AX-157257435	Glyma.02G244000	2	43200089..43204490	K01915	glnA, GLUL;glutamine synthetase [EC:6.3.1.2]
AX-157257435	Glyma.02G244100	2	43222701..43224588	K07766	E3.6.1.52;diphosphoinositol-polyphosphate diphosphatase [EC:3.6.1.52]
AX-157170687	Glyma.03G013200	3	1337139..1343939	K12614	DDX6, RCK, DHH1;ATP-dependent RNA helicase DDX6/DHH1 [EC:3.6.4.13]
AX-157170687	Glyma.03G013600	3	1379993..1390392	K15440	TAD1, ADAT1;tRNA-specific adenosine deaminase 1 [EC:3.5.4.34]
AX-157170687	Glyma.03G014300	3	1427595..1433362	K15095	E1.1.1.208;(+)-neomenthol dehydrogenase [EC:1.1.1.208]
AX-157407598	Glyma.03G188500	3	39957460..39962189	K13126	PABPC;polyadenylate-binding protein
AX-157407598	Glyma.03G189300	3	40021744..40024023	K02896	RP-L24e, RPL24;large subunit ribosomal protein L24e
AX-157407598	Glyma.03G189700	3	40049277..40056481	K00873	PK, pyk;pyruvate kinase [EC:2.7.1.40]
AX-157442983	Glyma.04G076700	4	6445919..6452821	K13201	TIA1, TIAL1;nucleolysin TIA-1/TIAR
AX-157442983	Glyma.04G077400	4	6499898..6503759	K02738	PSMB6;20S proteasome subunit beta 1 [EC:3.4.25.1]
AX-157442983	Glyma.04G078200	4	6541151..6546684	K03036	PSMD11, RPN6;26S proteasome regulatory subunit N6
AX-157126994	Glyma.06G310200	6	49870537..49873394	K13280	SEC11, sipW;signal peptidase I [EC:3.4.21.89]
AX-157126994	Glyma.06G310400	6	49880338..49884322	K11271	DSCC1, DCC1;sister chromatid cohesion protein DCC1
AX-157525995	Glyma.06G321900	6	51040796..51041580	K08905	psaG;photosystem I subunit V
AX-157525995	Glyma.06G322300	6	51058001..51065788	K14945	QKI;protein quaking
AX-157525995	Glyma.06G323000	6	51103666..51106094	K02868	RP-L11e, RPL11;large subunit ribosomal protein L11e
AX-157395672	Glyma.07G202300	7	37166660..37169027	K13257	CYP93C;2-hydroxyisoflavanone synthase [EC:1.14.14.87]
AX-157395672	Glyma.07G202500	7	37177013..37177959	K11253	H3;histone H3
AX-157395672	Glyma.07G204400	7	37336384..37340546	K00721	DPM1;dolichol-phosphate mannosyltransferase [EC:2.4.1.83]
AX-157388275	Glyma.08G345200	8	46015608..46023940	K06126	COQ6;ubiquinone biosynthesis monooxygenase Coq6 [EC:1.14.13.-]
AX-157388275	Glyma.08G346000	8	46083151..46086614	K03109	SRP9;signal recognition particle subunit SRP9
AX-157388275	Glyma.08G346500	8	46138146..46140731	K02995	RP-S8e, RPS8;small subunit ribosomal protein S8e

AX-157555217	Glyma.08G365900	8	47673091..47677260	K01809	manA, MPI;mannose-6-phosphate isomerase [EC:5.3.1.8]
AX-157555217	Glyma.08G367400	8	47783600..47785134	K17781	TIM13;mitochondrial import inner membrane translocase subunit TIM13
AX-157129457	Glyma.09G179500	9	40420404..40427085	K14376	PAP;poly(A) polymerase [EC:2.7.7.19]
AX-157480162	Glyma.10G279900	10	50130564..50136128	K02729	PSMA5;20S proteasome subunit alpha 5 [EC:3.4.25.1]
AX-157369770	Glyma.11G231000	11	32666235..32668355	K02865	RP-L10Ae, RPL10A;large subunit ribosomal protein L10Ae
AX-157090659	Glyma.14G010200	14	788305..792206	K12872	RBM22, SLT11;pre-mRNA-splicing factor RBM22/SLT11
AX-157090659	Glyma.14G010400	14	802172..805618	K02259	COX15, ctaA;cytochrome c oxidase assembly protein subunit 15
AX-157090659	Glyma.14G011600	14	887156..890657	K04079	HSP90A, htpG;molecular chaperone HtpG
AX-157439837	Glyma.15G152000	15	12592655..12594969	K09286	EREBP;EREBP-like factor
AX-157439837	Glyma.15G153300	15	12725123..12726992	K15032	MTERFD;mTERF domain-containing protein, mitochondrial
AX-157439837	Glyma.15G153400	15	12730592..12733439	K15032	MTERFD;mTERF domain-containing protein, mitochondrial
AX-157069540	Glyma.16G213300	16	37138811..37144846	K07151	STT3;dolichyl-diphosphooligosaccharide---protein glycosyltransferase [EC:2.4.99.18]
AX-157069540	Glyma.16G215500	16	37284766..37290393	K03245	EIF3J;translation initiation factor 3 subunit J
AX-157553398	Glyma.18G284200	18	56482736..56486831	K03247	EIF3H;translation initiation factor 3 subunit H
AX-157553398	Glyma.18G284300	18	56489793..56496952	K01634	SGPL1, DPL1;sphinganine-1-phosphate aldolase [EC:4.1.2.27]
AX-157268182	Glyma.18G286200	18	56637471..56646826	K12373	HEXA_B;hexosaminidase [EC:3.2.1.52]
AX-157268182	Glyma.18G286400	18	56665160..56667323	K02893	RP-L23Ae, RPL23A;large subunit ribosomal protein L23Ae
AX-157352830	Glyma.18G287400	18	56740754..56748259	K00164	OGDH, sucA;2-oxoglutarate dehydrogenase E1 component [EC:1.2.4.2]
AX-157352830	Glyma.18G287600	18	56754676..56759595	K07901	RAB8A, MEL;Ras-related protein Rab-8A
AX-157467282	Glyma.20G193100	20	43207414..43208918	K02933	RP-L6, MRPL6, rplF;large subunit ribosomal protein L6
AX-116884145	Glyma.20G240000	20	47137688..47144012	K12373	HEXA_B;hexosaminidase [EC:3.2.1.52]
AX-116884145	Glyma.20G240500	20	47172307..47177872	K12830	SF3B3, SAP130, RSE1;splicing factor 3B subunit 3
AX-116884145	Glyma.20G240700	20	47188810..47190879	K02918	RP-L35e, RPL35;large subunit ribosomal protein L35e
AX-116884145	Glyma.20G240800	20	47192875..47195439	K01728	pel;pectate lyase [EC:4.2.2.2]
AX-157324617	Glyma.20G184400	20	42259262..42264549	K12736	PPWD1;peptidylprolyl isomerase domain and WD repeat-containing protein 1 [EC:5.2.1.8]
AX-157324617	Glyma.20G185700	20	42428886..42431148	K13081	LAR;leucoanthocyanidin reductase [EC:1.17.1.3]

TABLES7 | Details of 129 genes with high expression in seeds.

Marker	Gene name	Chr	Position(bp)	KO number	FPKM
AX-116905453	Glyma.02G254500	2	44151303..44155647	K12947	52.688
AX-116905453	Glyma.02G255000	2	44207467..44209844	K09872	217.976
AX-116905453	Glyma.02G255500	2	44240358..44242714	K02941	165.212
AX-116905453	Glyma.02G255900	2	44276135..44279880		0.802
AX-157257435	Glyma.02G243800	2	43183707..43187668		7.728
AX-157257435	Glyma.02G244000	2	43200089..43204490	K01915	24.487
AX-157257435	Glyma.02G244100	2	43222701..43224588	K07766	12.43
AX-157257435	Glyma.02G244700	2	43291483..43294331		29.123
AX-157257435	Glyma.02G245000	2	43322226..43323956		33.388
AX-157257435	Glyma.02G245100	2	43327594..43329339	K12614	54.21
AX-157170687	Glyma.03G013200	3	1337139..1343939		22.07
AX-157170687	Glyma.03G013600	3	1379993..1390392	K15440	2.62
AX-157170687	Glyma.03G014300	3	1427595..1433362	K15095	34.775
AX-157170687	Glyma.03G014800	3	1493449..1494342		3.585
AX-157052803	Glyma.03G016700	3	1683395..1688893		14.006
AX-157052803	Glyma.03G016900	3	1702419..1707451		0.636
AX-157052803	Glyma.03G018200	3	1805212..1805754		0.741
AX-157052803	Glyma.03G018300	3	1819158..1819700		1.109
AX-157407598	Glyma.03G188400	3	39948670..39954592		10.853
AX-157407598	Glyma.03G188500	3	39957460..39962189	K13126	9.019
AX-157407598	Glyma.03G189000	3	40000792..40003958		1.852
AX-157407598	Glyma.03G189100	3	40010082..40013954		93.017
AX-157407598	Glyma.03G189300	3	40021744..40024023	K02896	95.385

AX-157407598	Glyma.03G189500	3	40038404..40042482		29.792
AX-157407598	Glyma.03G189700	3	40049277..40056481	K00873	16.416
AX-157407598	Glyma.03G189900	3	40073618..40076667		81.69
AX-157407598	Glyma.03G190000	3	40080041..40082648		9.892
AX-157407598	Glyma.03G190100	3	40086278..40087033		3.868
AX-116924879	Glyma.03G040400	3	5050463..5052307		288.52
AX-157528951	Glyma.03G048100	3	6183950..6185451		0.268
AX-157528951	Glyma.03G048400	3	6234529..6236350		0.165
AX-157442983	Glyma.04G076700	4	6445919..6452821	K13201	25.135
AX-157442983	Glyma.04G077200	4	6485571..6488561		0.516
AX-157442983	Glyma.04G077400	4	6499898..6503759	K02738	57.665
AX-157442983	Glyma.04G077700	4	6513126..6516245		14.248
AX-157442983	Glyma.04G078200	4	6541151..6546684	K03036	63.25
AX-157126994	Glyma.06G310200	6	49870537..49873394	K13280	88.504
AX-157126994	Glyma.06G310400	6	49880338..49884322	K11271	3.454
AX-157525995	Glyma.06G321400	6	51001786..51006353		13.713
AX-157525995	Glyma.06G321900	6	51040796..51041580	K08905	75.16
AX-157525995	Glyma.06G322100	6	51046118..51047447		590.436
AX-157525995	Glyma.06G322200	6	51048637..51055248		11.954
AX-157525995	Glyma.06G322300	6	51058001..51065788	K14945	41.91
AX-157525995	Glyma.06G322600	6	51075762..51080841		4.733
AX-157525995	Glyma.06G323000	6	51103666..51106094	K02868	115.337
AX-157525995	Glyma.06G323900	6	51161239..51165831		35.624
AX-157457247	Glyma.07G038400	7	3169310..3172002		26.871
AX-157395672	Glyma.07G202300	7	37166660..37169027	K13257	36.886
AX-157395672	Glyma.07G202500	7	37177013..37177959	K11253	25.07

AX-157395672	Glyma.07G202800	7	37195139..37199755		1.878
AX-157395672	Glyma.07G203000	7	37213013..37215962		0.531
AX-157395672	Glyma.07G204400	7	37336384..37340546	K00721	12.947
AX-157388275	Glyma.08G344800	8	45973271..45983627		8.394
AX-157388275	Glyma.08G345100	8	46005838..46007238		52.929
AX-157388275	Glyma.08G345200	8	46015608..46023940	K06126	20.459
AX-157388275	Glyma.08G346000	8	46083151..46086614	K03109	24.335
AX-157388275	Glyma.08G346100	8	46093776..46094633		1.765
AX-157388275	Glyma.08G346500	8	46138146..46140731	K02995	88.747
AX-157388275	Glyma.08G346600	8	46144080..46145206		751.588
AX-157555217	Glyma.08G365500	8	47641516..47643779		32.56
AX-157555217	Glyma.08G365700	8	47659642..47662878		9.471
AX-157555217	Glyma.08G365900	8	47673091..47677260	K01809	26.722
AX-157555217	Glyma.08G367400	8	47783600..47785134	K17781	23.22
AX-157555217	Glyma.08G367800	8	47807547..47810213		0.883
AX-157129457	Glyma.09G179300	9	40402524..40409805		24.491
AX-157129457	Glyma.09G179500	9	40420404..40427085	K14376	12.004
AX-157129457	Glyma.09G180400	9	40549388..40551670		10.852
AX-157129457	Glyma.09G180500	9	40552093..40553948		0.523
AX-157129457	Glyma.09G181100	9	40604855..40605934		61.282
AX-157355369	Glyma.10G270200	10	49255968..49258602		27.849
AX-157355369	Glyma.10G271000	10	49322674..49323913		43.522
AX-157480162	Glyma.10G279300	10	50085065..50091502		39.475
AX-157480162	Glyma.10G279400	10	50098784..50110697		4.763
AX-157480162	Glyma.10G279500	10	50111977..50114967		1.547
AX-157480162	Glyma.10G279900	10	50130564..50136128	K02729	40.587

AX-157480162	Glyma.10G280000	10	50139642..50146154		26.986
AX-157480162	Glyma.10G280100	10	50140759..50141070		1.526
AX-157480162	Glyma.10G280200	10	50143105..50143896		0.454
AX-157480162	Glyma.10G280400	10	50157107..50159056		8.07
AX-157480162	Glyma.10G280600	10	50174484..50178762		8.111
AX-157480162	Glyma.10G280800	10	50201731..50204631		25.578
AX-157480162	Glyma.10G280900	10	50205862..50212349		6.065
AX-157369770	Glyma.11G231000	11	32666235..32668355	K02865	226.913
AX-157369770	Glyma.11G231500	11	32716751..32717855		14.872
AX-157588392	Glyma.12G140100	12	17229831..17232009		130.167
AX-157090659	Glyma.14G010200	14	788305..792206	K12872	21.499
AX-157090659	Glyma.14G010400	14	802172..805618	K02259	9.205
AX-157090659	Glyma.14G010700	14	820330..820963		81.468
AX-157090659	Glyma.14G011400	14	863299..863760		1.11
AX-157090659	Glyma.14G011600	14	887156..890657	K04079	77.306
AX-157090659	Glyma.14G012200	14	920087..926504		13.316
AX-157439837	Glyma.15G152000	15	12592655..12594969	K09286	134.32
AX-157439837	Glyma.15G153100	15	12710775..12713895		3.805
AX-157439837	Glyma.15G153200	15	12719862..12720422		2.939
AX-157439837	Glyma.15G153300	15	12725123..12726992	K15032	3.819
AX-157439837	Glyma.15G153400	15	12730592..12733439	K15032	1.445
AX-157069540	Glyma.16G213300	16	37138811..37144846	K07151	40.553
AX-157069540	Glyma.16G214400	16	37213408..37214296		0.26
AX-157069540	Glyma.16G215500	16	37284766..37290393	K03245	21.758
AX-157575017	Glyma.16G168500	16	32844099..32848267		1.819
AX-157553398	Glyma.18G283400	18	56423007..56426460		204.719

AX-157553398	Glyma.18G284100	18	56477698..56481601		36.118
AX-157553398	Glyma.18G284200	18	56482736..56486831	K03247	63.867
AX-157553398	Glyma.18G284300	18	56489793..56496952	K01634	26.74
AX-157553398	Glyma.18G284700	18	56542358..56545578		20.574
AX-157268182	Glyma.18G284300	18	56489793..56496952	K01634	26.74
AX-157268182	Glyma.18G284700	18	56542358..56545578		20.574
AX-157268182	Glyma.18G286100	18	56628728..56632642		21.776
AX-157268182	Glyma.18G286200	18	56637471..56646826	K12373	24.334
AX-157268182	Glyma.18G286400	18	56665160..56667323	K02893	41.093
AX-157352830	Glyma.18G287400	18	56740754..56748259	K00164	26.251
AX-157352830	Glyma.18G287500	18	56749390..56753241		0.902
AX-157352830	Glyma.18G287600	18	56754676..56759595	K07901	34.701
AX-157352830	Glyma.18G288100	18	56791325..56794532		0.29
AX-157352830	Glyma.18G288400	18	56812346..56817728		0.742
AX-157352830	Glyma.18G289000	18	56863534..56867736		120.058
AX-157467282	Glyma.20G192500	20	43138131..43145708		8.552
AX-157467282	Glyma.20G193100	20	43207414..43208918	K02933	21.962
AX-116884145	Glyma.20G240000	20	47137688..47144012	K12373	14.762
AX-116884145	Glyma.20G240300	20	47159346..47162695		332.695
AX-116884145	Glyma.20G240500	20	47172307..47177872	K12830	9.136
AX-116884145	Glyma.20G240700	20	47188810..47190879	K02918	428.24
AX-116884145	Glyma.20G240800	20	47192875..47195439	K01728	7.455
AX-116884145	Glyma.20G241100	20	47224329..47228932		0.404
AX-157324617	Glyma.20G184400	20	42259262..42264549	K12736	27.79
AX-157324617	Glyma.20G184600	20	42279513..42280994		4.427
AX-157324617	Glyma.20G185100	20	42342266..42345129		14.541

AX-157324617	Glyma.20G185600	20	42416343..42426466		6.953
AX-157324617	Glyma.20G185700	20	42428886..42431148	K13081	9.469

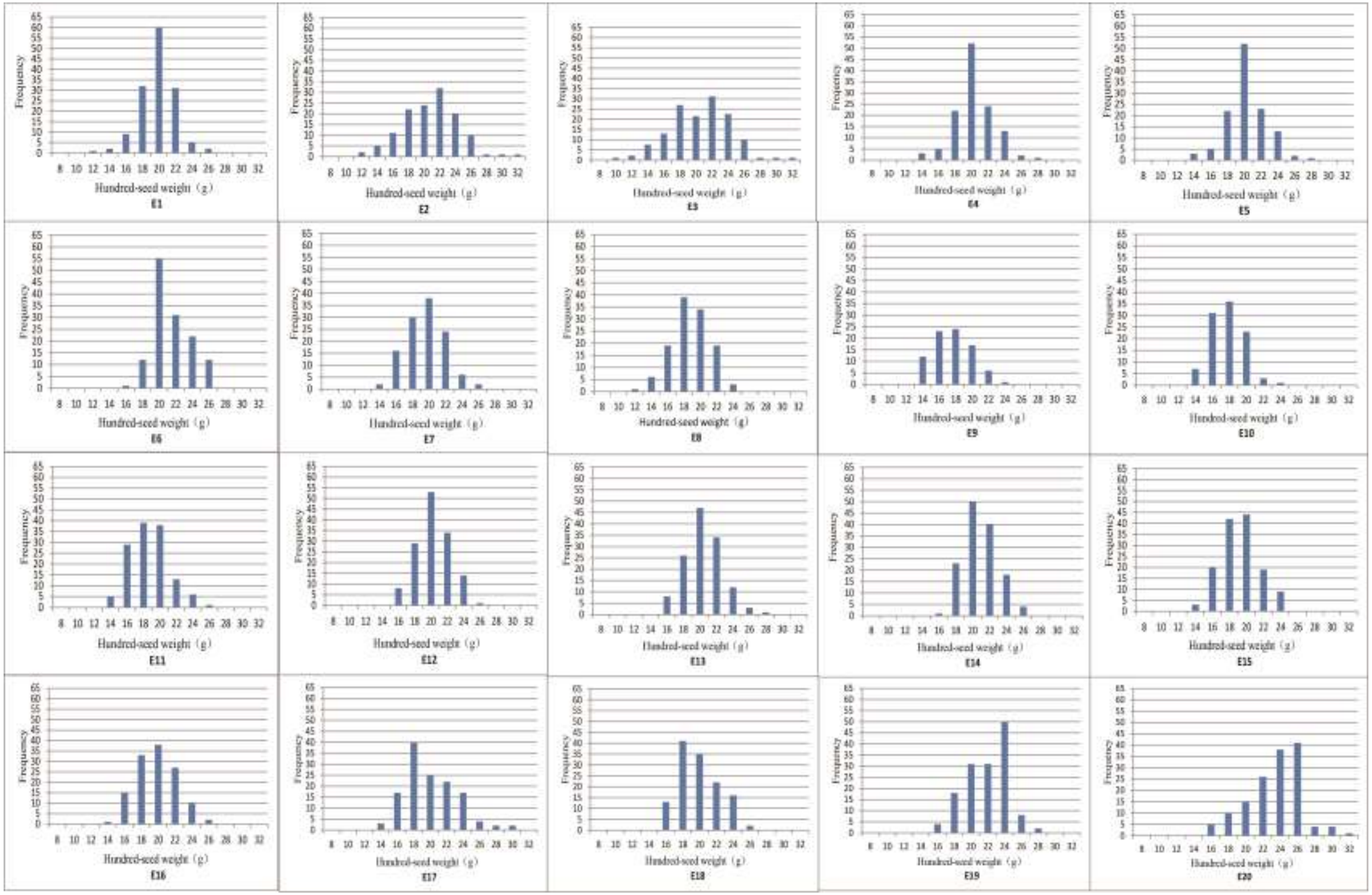
Frequency
Hundred-seed weight (g)
E1
E2
E3
E4
E5
E6
E7
E8
E9
E10
E11
E12
E13
E14
E15
E16
E17
E18
E19
E20

Figure S1 | Frequency distribution of 100-seed weight under 20 environments.
